# Meta-Analyses of Microarray Datasets Identifies *ANO1* and *FADD* as Prognostic Markers of Head and Neck Cancer

**DOI:** 10.1371/journal.pone.0147409

**Published:** 2016-01-25

**Authors:** Ram Bhupal Reddy, Anupama Rajan Bhat, Bonney Lee James, Sindhu Valiyaveedan Govindan, Rohit Mathew, Ravindra DR, Naveen Hedne, Jeyaram Illiayaraja, Vikram Kekatpure, Samanta S. Khora, Wesley Hicks, Pramila Tata, Moni A. Kuriakose, Amritha Suresh

**Affiliations:** 1 Integrated Head and Neck Oncology Program, Mazumdar Shaw Centre for Translational Research, Mazumdar Shaw Medical Centre, Narayana Health, Bangalore, Karnataka, India; 2 Head and Neck Oncology, Mazumdar Shaw Medical Centre, Narayana Health, Bangalore, Karnataka, India; 3 Division of Medical Biotechnology, School of Biosciences and Technology, Vellore Institute of Technology University, Vellore, Tamil Nadu, India; 4 Strand Life Sciences, Kirloskar Business Park, Bangalore, Karnataka, India; 5 Department of Clinical Research, Mazumdar Shaw Medical Centre, Narayana Health, Bangalore, Karnataka, India; 6 Department of Head and Neck/Plastic & Reconstructive Surgery, Roswell Park Cancer Institute, Buffalo, New York, United States of America; 7 Mazumdar Shaw Medical Centre-Roswell Park Collaboration Program, Roswell Park Cancer Institute, Buffalo, New York, United States of America; Queen Mary University of London, UNITED KINGDOM

## Abstract

The head and neck squamous cell carcinoma (HNSCC) transcriptome has been profiled extensively, nevertheless, identifying biomarkers that are clinically relevant and thereby with translational benefit, has been a major challenge. The objective of this study was to use a meta-analysis based approach to catalog candidate biomarkers with high potential for clinical application in HNSCC. Data from publically available microarray series (N = 20) profiled using Agilent (4X44K G4112F) and Affymetrix (HGU133A, U133A_2, U133Plus 2) platforms was downloaded and analyzed in a platform/chip-specific manner (GeneSpring software v12.5, Agilent, USA). Principal Component Analysis (PCA) and clustering analysis was carried out iteratively for segregating outliers; 140 normal and 277 tumor samples from 15 series were included in the final analysis. The analyses identified 181 differentially expressed, concordant and statistically significant genes; STRING analysis revealed interactions between 122 of them, with two major gene clusters connected by multiple nodes (MYC, FOS and HSPA4). Validation in the HNSCC-specific database (N = 528) in The Cancer Genome Atlas (TCGA) identified a panel (*ECT2*, *ANO1*, *TP63*, *FADD*, *EXT1*, *NCBP2*) that was altered in 30% of the samples. Validation in treatment naïve (Group I; N = 12) and post treatment (Group II; N = 12) patients identified 8 genes significantly associated with the disease (Area under curve>0.6). Correlation with recurrence/re-recurrence showed *ANO1* had highest efficacy (sensitivity: 0.8, specificity: 0.6) to predict failure in Group I. *UBE2V2*, *PLAC8*, *FADD* and *TTK* showed high sensitivity (1.00) in Group I while *UBE2V2* and *CRYM* were highly sensitive (>0.8) in predicting re-recurrence in Group II. Further, TCGA analysis showed that *ANO1* and *FADD*, located at 11q13, were co-expressed at transcript level and significantly associated with overall and disease-free survival (*p*<0.05). The meta-analysis approach adopted in this study has identified candidate markers correlated with disease outcome in HNSCC; further validation in a larger cohort of patients will establish their clinical relevance.

## Introduction

Molecular profiling of tumors, including Head and Neck Squamous Cell Carcinoma (HNSCC), has been employed to understand the mechanisms of carcinogenesis as well as the underlying reasons for treatment resistance. The increasing global incidence of HNSCC along with the lack of significant improvement in overall survival rate during the past four decades, necessitate newer approaches to understand the molecular mechanisms of the disease and to identify potential markers for early detection, prognosis and as targets for therapy. Molecular profiling in combination with patient validation in cancers of the prostate, ovary and breast has led to clinical application of marker panels such as Mamma print and Oncotype Dx.[1, 2]. Similar attempts to establish diagnostic or prognostic molecular markers in HNSCC for clinical application have remained elusive.

High throughput molecular profiling using techniques that catalog the differences at the whole genome, transcriptome [3] [4] [5] and proteome [6] levels have been carried out in HNSCC. These studies have catalogued markers of progression [7] [8] and response to treatment [9]. Nevertheless, translation of these markers for clinical benefit has not been achieved due to the challenges associated with high throughput techniques in terms of marker selection and the need for large scale patient validation. These challenges are further confounded due to issues such as significant discordance between different high throughput studies, attributed to differences in sample processing, type of platforms used and the analytical algorithms applied [10] [11]. This problem is further augmented in transcriptomic studies primarily because transcript level changes can vary based on stage, tissue-specific and spatial fluctuations in expression of various genes.

Analytical strategies that can utilize existing databases and identify markers concordant across studies may be a beneficial approach to narrow down to markers of increased confidence and clinical applicability. Meta—analysis, referring to the analysis of publically available datasets, could hence, potentially enable identification of clinically relevant pool of markers; many such studies have been reported in cancers of different sites. Meta-analysis based approaches in pancreatic cancer have led to identification of novel targets and candidate biomarkers [12]. In prostate cancers, markers causal to the carcinogenic process were identified using a similar approach [13]. In addition, markers highly associated with prognosis and treatment outcome prediction in breast cancer [14] [15] were also identified through similar meta-analysis based approaches. The primary objective of this study was to carry out a cross-platform, cross-study meta-analysis of publically available microarray datasets in head and neck cancer, identify markers of biological relevance through a common analytical pipeline and subsequently validate them in clinical samples.

## Materials and Methods

### Search Criteria and Data Mining

**Fig 1:**
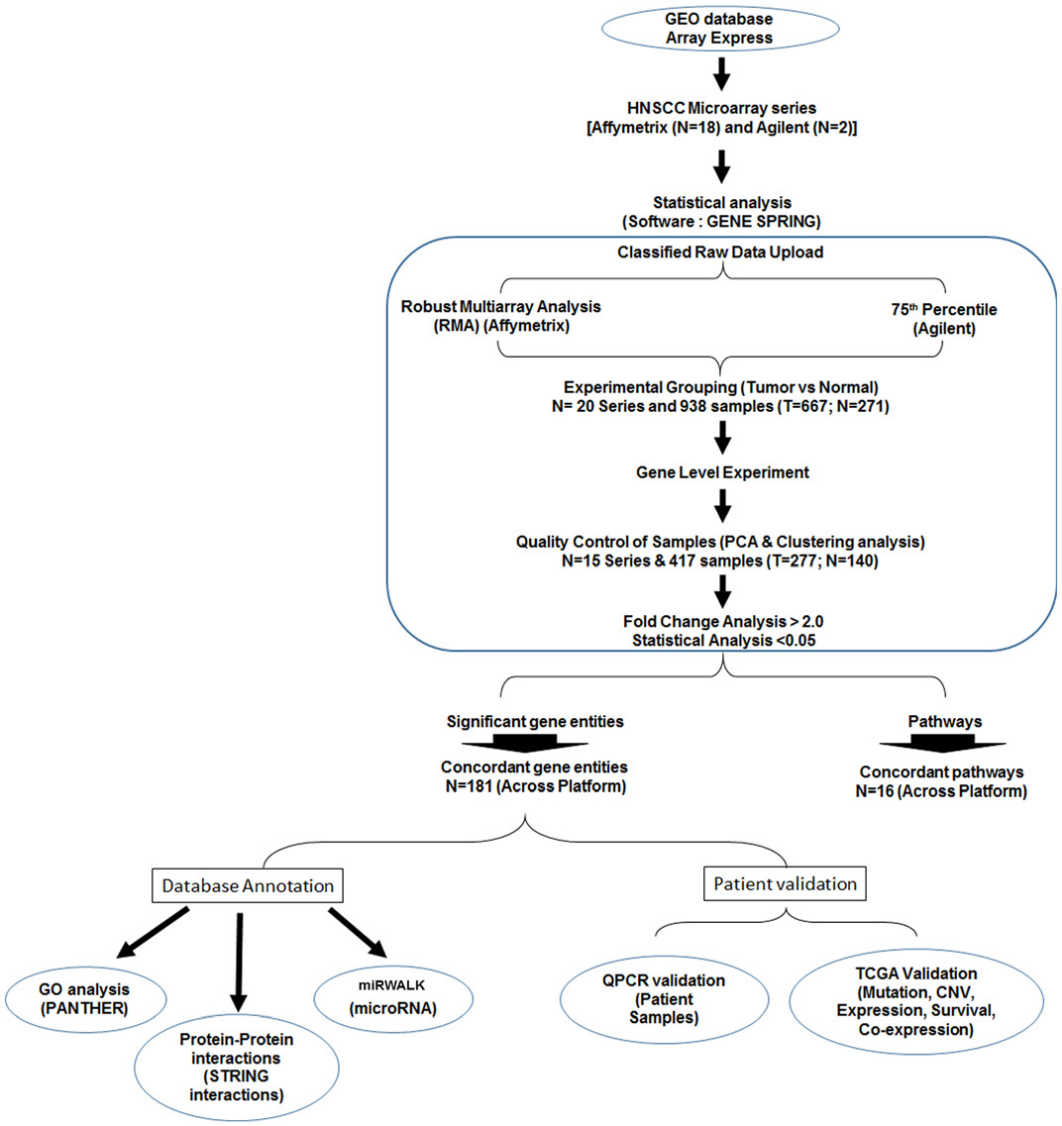
Meta-analysis work flow. The publically available raw microarray data of Head and Neck Squamous Cell Carcinoma (HNSCC) series were downloaded and grouped and analyzed in Genespring statistical software. Normalization was carried out on the samples, which were then grouped into tumor and normal prior to performing the gene level experiment. Principal Component Analysis (PCA) was performed to remove the discordant samples. Fold change and *p* value were calculated to obtain the significant gene entities. These statistically significant entities were used for further analysis; database annotations (Gene Ontology, STRING and miRWALK) and patient validation (Experimental validation by Quantitative Real Time PCR (qPCR) and The Cancer Genome Atlas (TCGA)).

Analysis of the publically available microarray datasets was carried out as per the PRISMA guidelines [16]. The public databases, Gene Expression Omnibus (GEO) (NCBI—http://www.ncbi.nlm.nih.gov/geo/) and Array Express (EBI) [http://www.ebi.ac.uk/arrayexpress] were searched for the presence of raw data of microarray experiments carried out in head and neck cancer. The series selected for the analysis included data from i) studies carried out in Head and Neck Squamous Cell carcinoma patients ii) studies including treatment naïve patients and iii) studies carrying out global profiling of transcriptomics using high-density arrays. Studies/samples including thyroid, oropharynx and nasopharynx were excluded from the study due to their varied etiologies. The two platforms included in the analyses were Affymetrix [Affymetrix Inc., California, USA] and Agilent [Agilent Technologies, California, USA]. The raw data series profiled by both the platforms were grouped based on the individual technology and each technology (of either platform) was included in the analysis if at least 2 series were available in the database. The general work flow followed the stepwise protocol suggested by Ramasamy *et al* for carrying out meta-analysis [17]. The basic work algorithm is detailed in Fig 1.

### Data Analysis Using Gene Spring

The raw data files used for the analyses included .CEL (Affymetrix platform) and .TXT (Agilent) files. Meta-analysis was carried out using Gene spring software (http://genespring-support.com) [v12.5, Agilent, California, USA]. The raw data pertaining to each chip was uploaded onto the software wherein the samples were baseline transformed and normalized by Robust Multi-array Analysis (RMA) in Affymetrix or 75^th^ percentile in Agilent platforms (single color). The individual sample files were then classified into ‘normal’ and ‘tumor’ and re-analyzed as a single experiment. Gene level experimental data (arithmetic mean of all probes mapping to the same probe ID) was generated and quality control was carried out using Principal Component Analysis (PCA) in GeneSpring. The samples that were outliers in the PCA plots were removed and clustering carried out subsequently to ensure a clear stratification between the two categories of samples (Normal and Tumor). The process was carried out with multiple iterations to obtain a refined separation. Fold change analysis was then executed on the samples following which unpaired *t*-test (unequal variance) was performed to obtain significant gene entities. The *p*-value computation (asymptotic) and multiple testing correction (Benjamini Hochberg FDR) were further performed to obtain gene entities with *p*-value <0.05 and fold change (FC) of > 2.0. Inbuilt pathway analysis was carried out using the identified gene list. The significant gene list and pathways across the different technologies of Affymetrix were compared using the gene symbol/pathway names as an identifier. The individual gene entity list from each technology were extracted from the Genespring software and exported to excel files. The concordant gene entities as well as common pathways were identified across the technologies by using Microsoft Access (Microsoft Inc. WA, USA). Similar approach was adopted to identify all the common genes and pathways across the two platforms of Affymetrix and Agilent (Fig 1).

### Functional Annotation Using Multiple Web Resources

The concordant gene list across the different platforms was analyzed in the different web resources to assess functional classes, protein-protein interactions and miRNAs targeting the genes. Gene Ontology Analysis was performed using PANTHER (Protein Annotation through Evolutionary Relationship) classification system (http://www.pantherdb.org/) [18] to evaluate the functional classes of the genes. The STRING database (STRING v9.1) (http://string-db.orgnewstring_cgi) [19] was used to predict and catalog the protein-protein interactions between the concordant genes. The miRNAs that target these genes were investigated using the miRWalk2.0 database (http://www.umm.uni-heidelberg.de/apps/zmf/mirwalk/index.html) [20].

### Patient Based Validation

The concordant gene list was also cross compared with the TCGA database (http://www.cbioportal.org) [21] for their mutation, copy number variation (CNV) and mRNA expression status. The significant gene panel identified was also assessed for their co-expression data, overall and disease-free survival in correlation with their expression patterns in the TCGA cancer studies (Head and Neck Squamous cell carcinoma, Provisional; N = 528 samples).

The validation of selected genes was also carried out in HNSCC patients using quantitative real time PCR. The study was approved by Narayana Hrudayalaya Hospitals Institutional review board [NH/IRB-CL-2012-058]. Surgical samples of patients were selected from the bio repository of the Head and Neck Oncology department, Mazumdar Shaw Medical Center, Bangalore. All samples were collected after written informed consent. Previously untreated and recurrent patients diagnosed with HNSCC of all sites (excluding thyroid, oropharynx and nasopharynx) and stages during the period March 2010 to 2014, with details of follow up, were included in the study. The normal tissue samples were collected from healthy volunteers during the dental extraction after written informed consent. The demographic, clinical and pathological data of the patients were obtained from the electronic medical records and the patients were followed up for a period of 2 years.

The patients were categorized based on their Human papillomavirus (HPV) status as identified by p16 immunohistochemistry using established protocols. Formalin fixed paraffin embedded sections of the patients were collected from the department of pathology at the center. Briefly, tumor sections (5 micron) were de-paraffinised and treated with 3% hydrogen peroxide to stop endogenous peroxidase activity. The sections were incubated with the primary antibody (anti-p16, BioGenex, Fremont, CA, USA) and subsequent detection was carried out using the Horse Radish Peroxidase (HRP) system as per manufacturer’s instructions (Dako REAL^™^ EnVision^™^ Detection System, Denmark). Sections processed similarly but without the primary antibody were taken as negative controls. The scoring of the stained slides was as per established studies [22]. The slides were evaluated using light microscopy and scored on a 0–3 scale taking into account percentage positivity and intensity of staining; 0 (complete absence of tumor staining), 1 (weak staining of tumor cells), 2 (< 50% tumors cells stained with moderate intensity) and 3 (>50% tumor staining with moderate/intense staining).

### Marker Profiling for Validation by Quantitative Real Time PCR (qPCR)

RNA was extracted from the samples, archived in RNA Later (Ambion, Thermo Fisher Scientific, Massachusetts, USA), using TRIZOL reagent (Sigma Aldrich, MO, USA) and treated with DNase (Fermentas, Thermo Fisher Scientific, Massachusetts, USA) and assessed for contamination by Polymerase Chain Reaction (PCR). The integrity and quality of RNA was ascertained by gel electrophoresis and the 260/280 ratio. 1μg of RNA (260/280: 1.8–2.0) was converted into complementary DNA by the High Capacity cDNA Conversion kit (Applied Biosystems, CA, USA) as per the manufacturer’s instructions. The selected markers were profiled using specific primers listed in S1 Table and all primers were evaluated for specificity using Basic Local Alignment Search Tool (BLAST) analysis (National Center for Biological Information, NLM, US). The qPCR efficiency was assessed using the slope of the linear regression model as per standard protocols. The Cp was measured across multiple serial dilutions of each cDNA to obtain the standard curve and the corresponding slope. The efficiency is calculated using the formula, Efficiency = 10^(-1/slope)^. All real time PCR reactions were performed in triplicates using the Roche Light Cycler 480 real time PCR system (Roche Diagnostics, Germany) or the ABI Step One Real Time Machine (Applied Biosystems, CA, USA) using the Kapa SYBR Green PCR Master Mix (Kapa Biosystems, MA, USA). The thermal cycling conditions were 50°C for 2 min followed by an initial denaturation step at 95°C for 10 min, 45 cycles at 95°C for 30s, 60°C for 30s and 72°C for 30s. The experiments were carried out in triplicate for each data point. The Crossing point (Cp) and the subsequent analysis was carried out using the Second derivative max method in the software (Roche Diagnostics, Basel, Switzerland). All reactions were confirmed for their specificity by melting curve analysis of sample and the no-target control (NTC). A set (N = 4) of reference genes [(Glyceraldehyde-3-Phosphate Dehydrogenase (*GAPDH*), 60S acidic ribosomal protein P0 (*RPLP0*), 18S ribosomal Ribonucleic Acid (*18SRNA*) and β-Glucuronidase (*GUS*)] were assessed using the RefFinder [23] in a subset of the patient cohort (N = 26) and two of the best Reference Genes (RGs) were selected for further analysis. The relative change in expression was evaluated using the geometric mean of the selected reference genes [24]. The expression in normal samples served as the calibrator.

### Statistical Methods

Statistical analysis was performed using STATA11.1 (College Station, TX, USA). Receiver Operating characteristic (ROC) curve analysis was used to evaluate the predictive power of each of the biomarkers, the optimal cut point that yielded the maximum sensitivity and specificity was determined for each biomarker. ROC curves were then plotted on the basis of the set of optimal sensitivity and specificity values. Area under the curve (AUC) was computed via numerical integration of ROC Curves. The biomarker that has the largest area under the ROC curve was identified as having the strongest association with the presence of head and neck tumor. The sensitivity and specificity of the association of the markers with recurrence/re-recurrence was analyzed using Clinical calculator (http://vassarstats.net/clin1.html).

## Results

### Data Mining

Based on the search criteria, data mining of the different databases identified a total of 42 series [Affymetrix platform: N = 32, Agilent: N = 10]. After a further filtration of the data based on the inclusion and exclusion criteria, 18 series from Affymetrix and two from Agilent were included in the study (Table 1). The technology and chips that were incompatible with the analytical pipeline and wherein only one series was available were excluded from the study. The total number of samples analyzed were 667 tumors and 271 normal. The Affymetrix platform included a total of 246 normal and 629 tumors (U133 plus 2.0: N = 207, T = 419; U133A chip: N = 19, T = 147; U133A_2: N = 20, T = 63) while the Agilent platform (4X44K G4112F) included 25 Normal (N) and 38 tumor (T). The sub sites investigated in the selected series were buccal mucosa, tongue, larynx, pharynx, and alveolus and retro-molar trigone. The normal samples were from gingivobuccal site in all the series. The series from each chip within each platform were analyzed separately using Gene spring analysis software and as per the analytical pipeline to identify the concordant gene entities lists.

**Table 1:** Details of series used in the Meta-analysis.

S.NO	PUBLIC DATA SETS	ARRAY PLATFORM	SITE DETAILS*	GENES IDENTIFIED	GENES VALIDATED	VALIDATION METHOD	PUBMED ID
1	GSE51010	HG-U133_Plus_2	OSCC	-	-	-	-
2	GSE31287	HG-U133_Plus_2	HNSCC	-	-	-	PMID: 22234739
3	GSE10300	HG-U133_Plus_2	HNSCC	-	-	-	PMID: 19117988, PMID: 16467079,
4	GSE17913	HG-U133_Plus_2	BUCCAL MUCOSA	41	S100A7, CYP1B1, CYP1A1, CD207, CHRNA3, NQO1, PTGES, AHRR, CD1a, LEPR, IGF2BP3	qPCR, IHC, WESTERN BLOTTING	PMID: 20179299
5	GSE30784	HG-U133_Plus_2	OSCC	131	LAMC2, COL4A1, COL1A1, and PADI1	qPCR	PMID: 18669583
6	GSE3292	HG-U133_Plus_2	HNSCC	91	TAF7L, CDKN2A, SYCP2, RFC4, and NAP1L2	qPCR	PMID: 16943533, PMID: 16467079,
7	GSE9844	HG-U133_Plus_2	TONGUE	35	IL8 and MMP9	qPCR, IHC	PMID: 18254958
8	GSE6791	HG-U133_Plus_2	HNSCC, CERVICAL CANCER SAMPLES	-	SYCP2 and TCAM1	qPCR, IHC, WESTERN BLOT	PMID: 17510386
9	GSE7224	HG-U133_Plus_2	ORAL EPITHELIA, TONSIL	-	CXCR4, CCR5, CD19, CD3, defensin-β1, defensin-β4, SLPI, ICAM-3, CD4	IHC	PMID: 17620369
10	GSE9600	HG-U133_Plus_2	HNSCC	-	-	-	PMID: 20652976
11	GSE16149	HG-U133_Plus_2	BUCCAL MUCOSA	-	-	-	PMID: 20576139
12	GSE31056	HG-U133_Plus_2	TONGUE	139	MMP1, COL4A1, P4HA2, and THBS2	qPCR	PMID: 21989116
13	GSE45153	HG-U133_Plus_2	HNSCC	-	-	-	PMID: 23981300
14	GSE2280	HG-U133A	OSCC	116	CXCR4	qPCR, IHC	PMID: 15558013
15	GSE27020	HG-U133A	LARYNGEAL CANCER	30	ACE2, DHTKD1, FLOT1, MAP4K1, NEK2, SFRS8, PRKD1, TBC1D4, TGOLN2, YTHDC2,	qPCR	PMID: 23950933
16	GSE3524	HG-U133A	OSCC	53	LGALS1, MMP1, LAGY, and KRT4	qPCR	PMID: 15381369
17	GSE8987#	HG-U133A	BUCCAL MUCOSA, NASAL EPITHELIUM	314	CEACAM5, CYP4F11 and S100P	qPCR	PMID: 18513428
18	GSE23036	HG-U133A_2	HNSCC		RAB25, THBS1 and DUOX1	qPCR	PMID: 22696598
19	GSE8987#	HG-U133A_2	BUCCAL MUCOSA, NASAL EPITHELIUM	314	CEACAM5, CYP4F11 and S100P	qPCR	PMID: 18513428
20	GSE23558	Agilent-014850 / 4x44K G4112F	OSCC	315	SPP1, CA9, HOXC9, TNFRSF12A, LY6K, INHBA, FST, MFAP5 and DHRS2,MAL, TSN, SLC4A1AP, GPX3	qPCR, IHC	PMID: 22072328
21	GSE46802	Agilent-014850 / 4x44K G4112F	OSCC, NORMAL, DYSPLASIA	-	-	-	PMID: 24035722

* Only treatment Naïve and HNSCC samples were taken from these datasets for the analysis

^#^ This series has samples analyzed by two different technologies

OSCC: Oral Squamous Cell Carcinoma

IHC: Immunohistochemistry

#### Analysis across a single platform

In the Affymetrix platform, 18 series were analyzed, which included studies carried out on U133 plus 2 (N = 13), U133 A_2 (N = 2) and U133A (N = 4) chips; in one of the series, data was analyzed using two different chips (Table 1). Five series of U133 plus 2 were excluded since the samples were outliers during PCA and clustering; a total of 373 samples were included in the final analysis (N = 122, T = 251). The statistically significant gene list with a FC >2.0 and a *p*-value of <0.05 was used for further analysis. A total of 12,079 genes were identified after the analyses of U133 plus 2.0 while 4134 were identified from U133A and 3438 genes gene from U133A_2. Microsoft access analysis across these gene lists revealed a list of 965 gene entities of which 64.46% (N = 622) entities are concordant across the 3 chips (585 up regulated and 37 down regulated) (S1A File) and 35.54% (N = 343) of the genes showed mismatched trends of regulation. Similarly, an assessment of the concordance and discordance in regulation carried out across the technologies showed different trends (U133 Plus 2 Vs U133A Concordance = 76.53%; U133 Plus 2 Vs U133A_2 Concordance = 58.27%; U133A Vs U133A_2 Concordance = 86.11%).

The common pathways across the technologies were identified by comparison of their individual pathway lists. The list (N = 54) was then sorted based on the percentage of matched entities with respect to the total number of pathway entities. Twenty one pathways were identified with 30% or more matched entities in all the 3 technologies, among which a majority of them are cell cycle related pathways (S1B File).

In the Agilent platform (4x44k G4112F), 2 series, from which a total of 44 samples (N = 18, T = 26) were included in the final analysis A total of 8493 genes (up and down regulated) were identified from the analysis (FC>2.0; *p*<0.05); 338 of them being highly significant (*p*<0.001) with high fold change differences (FC>25 fold). Applying a cut off of 50% of matched entities from the total number of entities, 43 pathways were identified. The most significant pathways (> 65% of matched entities) identified were inflammatory response and extracellular matrix organization (ECM) (S1C and S1D File).

#### Cross-platform Analysis

The list of genes commonly identified between the two platforms revealed a total of 402 genes (*p*<0.05 and FC > 2.0) of which 181 (45.03%; 13 down regulated and 168 up regulated) were concordant and 221 (54.97%) discordant in the regulation trend. The concordant list of 181 genes is provided in the S2A File. Gene ontology analysis carried out for this gene list in the PANTHER database for the classification of the genes, indicated that in the molecular functions, the binding category (GO: 0005488) showed maximum representation (28.5%; N = 49) with protein and nucleic acid binding molecules being the major components. Similarly in biological processes, the cellular (GO: 0009987; 20.10%; N = 75) and metabolic process (GO: 0008152; 18.8%; N = 70) were a majority. 24.40% of the cellular components were cell parts (GO: 0043226) and extracellular regions (GO: 0005576). The majority of the protein classes identified were transporters (PC00227) (9.30%; N = 21) and receptors (PC00197) (8.40%; N = 8.) (S1A Fig).

The common pathways (N = 16) were identified across the two platforms with MS access. The most significant pathways identified were those associated with cytoskeletal behavior, cholesterol biosynthesis and IGF transport (>70% matched entities across the platforms). Oncostatin M signaling and Focal Adhesion were the other significant pathways that were dysregulated (>25% matched entities across the platforms) (S2B File).

**Fig 2:**
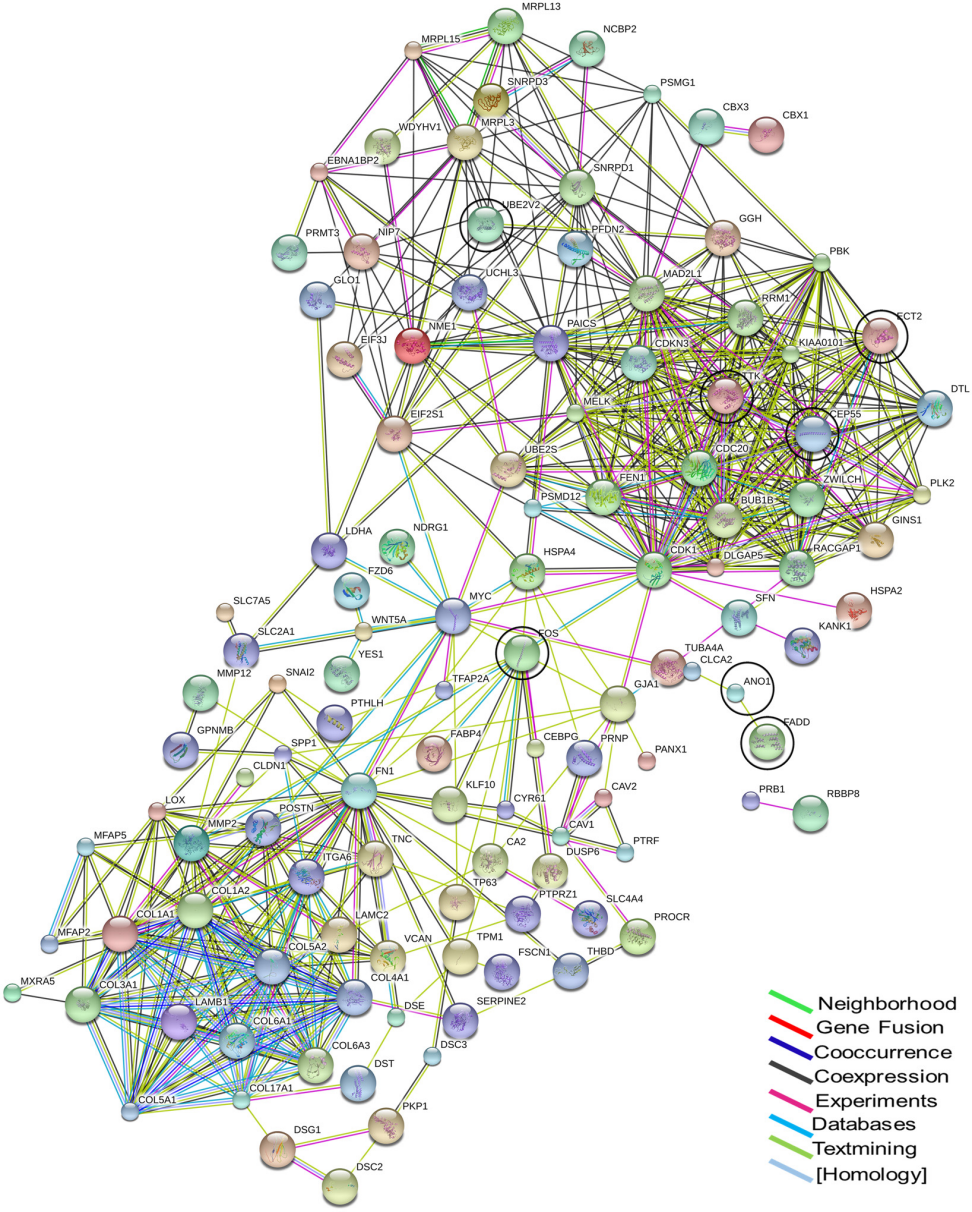
Identification of Protein-Protein Interaction. Analysis for protein-protein interaction by STRING network identified two major interconnecting clusters with high degree interactions between the genes (N = 122). These 2 major clusters were interconnected by the nodes MYC, FN1, FOS and HSPA4. The number of lines represent the levels of evidence as indicated in the color legend. The different sizes of the node are based on the extent of protein structural information available for each gene while the colors of the node are a visual aid used for better representation. The markers from this analysis selected for patient validation are encircled.

STRING database analyses identified a network of interactions between 122 genes from concordant list. The largest interaction network consisted of two large interconnected groups with FBJ Murine Osteosarcoma Viral Oncogene Homolog (FOS), Fibronectin 1 (FN1), Cyclin-Dependent Kinase 1 (CDK1), Heat shock 70 kDa protein 4 (HSPA4) and V-Myc Avian Myelocytomatosis (MYC) being the nodes of connection (Fig 2). The concordant list when further analyzed to identify the experimentally validated gene targets for miRNA expression from the miRWalk2.0 database, two major families of miRNA [*let* (N = 17) and *mir* (N = 47)] were identified, which targeted more than 5 genes from the list (S3 File).

### TCGA Database and Experimental Validation of the Genes in HNSCC Patients

The 181 gene list was cross compared with the TCGA database (http://www.cbioportal.org) to assess the mutation, copy number and mRNA expression status in the head and neck cancer cohort (N = 300). A total of 59 genes were altered in at least 10% of the patients while a sub-set of 6 genes [(Epithelial Cell Transforming gene 2 (*ECT2)*, Anoctamin 1 *(ANO1)*, Tumor Protein p63 *(TP63)*, Fas -Associated Via Death Domain *(FADD)*, Exostosin-1 *(EXT1)* and Nuclear Cap Binding Protein Subunit 2 *(NCBP2*)] were altered in at least 30% of the samples at the mutation/CNV and mRNA levels (S3 File and S1B Fig). The top 20 genes based on the percentage alterations in TCGA are listed in Table 2.

**Table 2:** List of top 20 genes based on the percentage alterations in TCGA.

S. No	GENE SYMBOL	Chromosome Location	Alteration in Gene Expression (N = 498)		Agilent		U133 PLUS 2		U133A		U133A_2	
			Number of Cases altered	%	p-value	FC (abs)	p-value	FC (abs)	p-value	FC (abs)	p-value	FC (abs)
1	FADD	11q13.3	161	32	0.000578044	2.08	2.65787E-11	5.49	2.28365E-10	2.12	1.36636E-10	2.55
2	ECT2	3q26.1-q26.2	146	29	8.42812E-12	4.27	3.40149E-21	3.92	1.4E-45	5.54	4.03399E-16	14.62
3	NCBP2	3q29	129	26	7.30899E-11	2.08	1.25625E-19	11.86	3.04538E-14	4.21	3.38459E-18	2.68
4	ANO1	11q13.3	122	24	4.76112E-09	17.91	1.57666E-06	2.78	4.68936E-20	3.33	1.27457E-21	15.26
5	EXT1	8q24.11	122	24	1.41097E-17	6.82	5.41787E-13	5.53	3.76146E-12	4.03	2.8362E-15	3.21
6	NDRG1	8q24.3	97	19	1.4748E-17	20.44	1.18762E-09	31.84	0.000021852	2.14	2.08484E-09	4.83
7	TP63	3q28	96	19	5.15547E-23	13.19	3.14883E-18	8.36	9.07143E-32	3.79	4.49354E-13	3.9
8	UBE2V2	8q11.21	87	17	2.45206E-12	3.72	1.1042E-12	10.24	6.6063E-09	4.7	7.18068E-10	5.61
9	KLF10	8q22.2	78	16	1.08314E-06	2.03	1.00163E-12	17.06	1.7561E-07	7.28	4.66826E-15	4.77
10	YES1	18p11.31-p11.21	77	15	1.40716E-12	5.28	3.07102E-09	3.28	1.62405E-05	2.85	2.87721E-07	2.38
11	MRPL3	3q21-q23	70	14	8.47739E-13	2.32	2.38555E-29	145.69	2.53596E-09	26.54	4.99042E-08	2.19
12	TRIB1	8q24.13	70	14	3.69935E-12	3.5	2.05752E-06	4.96	0.005448941	2.71	2.13018E-09	2.54
13	WDYHV1	8q24.13	70	14	6.85544E-07	2.13	1.13573E-06	3.97	4.51362E-13	2.28	1.40299E-08	3.13
14	EIF2S1	14q23.3	65	13	2.23584E-06	2.57	1.22031E-09	5.45	2.22462E-09	3.15	1.99808E-21	2.59
15	FSCN1	7p22	67	13	4.37413E-18	21.28	1.91554E-10	3.49	1.0799E-36	6.68	2.80616E-15	4.66
16	CLDN1	3q28-q29	61	12	0.02748958	2.65	3.61899E-38	48.07	4.25964E-10	2.17	0.001202747	2.07
17	FUBP3	9q34.11	59	12	7.18473E-14	2.19	4.82326E-13	5.29	1.59966E-18	4.67	1.92657E-07	3.26
18	SNAI2	8q11	59	12	9.67078E-17	17.59	1.61049E-19	23.56	6.20397E-17	14.27	3.2735E-14	14.92
19	GPR87	3q24	55	11	2.89582E-12	10.47	9.98395E-13	24.53	7.94468E-07	5.87	8.74107E-16	4.53
20	MRPL13	8q22.1-q22.3	56	11	9.44509E-15	2.94	6.90618E-18	25.09	6.74529E-18	8.11	6.46009E-09	2.56

Experimental validation using Quantitative Real time PCR (qPCR) was carried out in a total of 34 samples including primary untreated tumors (Group I; N = 12), recurrent samples (Group II; N = 12) and healthy normal controls (N = 10). Majority of the samples in the patient cohort (N = 24) were male (75%; N = 18) and from the oral cavity sub site (83.3%; N = 20) with advanced stage IV cancers (62.5%; N = 15). Majority of the patients were above the age of 40 yrs (95.8%; N = 23) and 79.2% were either smokers, chewers or alcohol consumers (N = 19). The patients in the primary cohort either underwent radiation after surgery or had no adjuvant treatment. Five of these patients developed recurrence during follow up while three were disease free (Four patients were lost to follow up) (S2 Table). In the recurrent cohort, the patients underwent chemotherapy and radiation prior to the surgical treatment.

The genes for validation were selected from the 181 gene list based on multiple criteria; down regulated more than 5 fold in 2 technologies [Placenta-Specific 8 (*PLAC8*) and Crystallin, Mu (*CRYM*)], nodes within the String database (*FOS*, *TTK*, Ubiquitin conjugating enzyme E2 variant 2 (*UBE2V2)* and Centrosomal Protein 55kDa (*CEP55*)] and subset of genes with alterations in at least 30% of patients (mutation, CNV and expression) (*ECT2*, *ANO1*, *FADD*) in TCGA database. All the primers, when validated showed an efficiency ranging from 1.9 to 2.1 with the percentage efficiency being 93% to 110% (S2 Fig). Analysis by RefFinder indicated that among the reference genes assessed, *RPLP0* and *18SRNA* were evaluated as the most stable (Geometric mean of ranking values: *RPLP0*: 1.19, *18SRNA*: 1.41, *GAPDH*: 3.0 and *GUS*: 4.00) after analysis by multiple software (Delta CT, geNORM, Normfinder and Bestkeeper) (S3 Fig). qPCR validation of the selected genes (N = 9) was then carried out using the relative quantification method using the geometric mean of the two reference genes.

**Fig 3:**
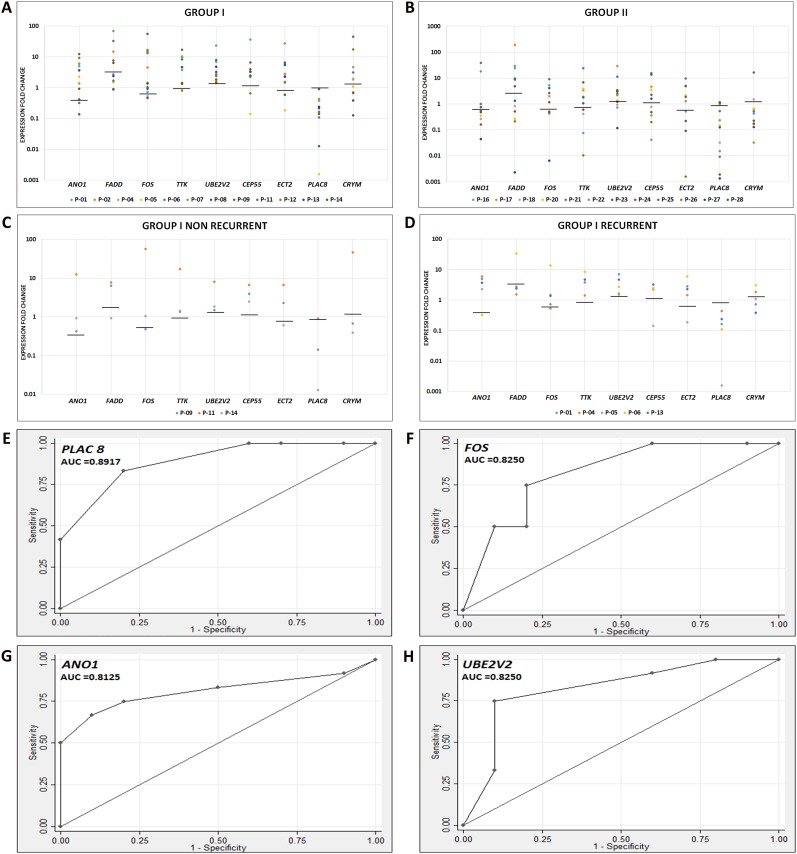
Validation of the markers in patients. Quantitative gene expression profiling of the selected markers was carried out in Group I (primary; A) and the Group II (recurrent; B) cohort. *PLAC8* and *UBE2V2* were validated in all the samples (100%) of Group I with regard to regulation trends whereas other genes showed similar trend in >60% of the samples. In Group II, >60% of the patients showed concordant regulation trends for four genes. Based on the patient follow-up, the Group I was sub-categorized into non-recurrent (C) and recurrent (D) and the expression was further evaluated. ROC curve analysis in the Group I patients showed that *PLAC8* (E), *FOS* (F), *ANO1* (G) and *UBE2V2* (H) had highest association with the disease (AUC >0.8). Bar represents the median fold change of Normals.

The markers showed regulatory trends in both the groups similar to those obtained from the meta-analysis (Fig 3A–3D). Six of the genes showed more than 70% validation in the samples assessed; *PLAC8* (91.67), *UBE2V2* (87.5%), *ECT2* (72.2%), *FADD* (69.5%), *CEP55* (69.5%) and *TTK* (76.2%). Assessment of the validation status in the two cohorts indicated that while all the genes were validated above 60% in Group I, only 4 genes showed a similar validation in Group II (*PLAC8*, *CRYM*, *ECT2* and *UBE2V2*). ROC analysis of the markers in the Cohort 1 indicated that *PLAC8* (AUC: 0.89), *FOS* (AUC 0.82), *ANO1* (AUC: 0.81) and *UBE2V2* (AUC: 0.82) had the highest association with the disease (Fig 3E–3H).

Assessment of HPV status, using p16 as the surrogate marker, indicated that among the patient cohort with available samples (N = 20), 12 patients had weak or no staining, while the rest had moderate/strong staining of p16 (N = 8) (S4A–S4D Fig). Correlation of the genes with the HPV status (with HPV scores of 2–3 taken as positive), indicated that the overall expression pattern of the markers does not show any association with HPV status. Individual analyses indicates that among the markers, only *FADD* showed an association with HPV positivity; increased percentage of patients (83%) with weak or no staining of HPV showed high expression of *FADD* as compared to patients positive for HPV (57%; p = 0.03) (S4E Fig).

### Correlation of the Marker Profile with Treatment Outcome

The markers were correlated with treatment outcome (recurrence in the Group I and re-recurrence in the Group II). *ANO1* showed the best efficacy (sensitivity: 0.83, specificity: 0.67) for detecting patients that fail treatment in cohort 1. *ANO1* also showed a 3-fold increase in median fold expression in the recurrent cohort (4/5) of Group I when compared to non-recurrent cohort. *ECT2* (0.83), *FADD* and *UBE2V2* (1) also showed high sensitivity in Group I. In Group II 7/9 (*UBE2V2*, *CRYM*, *FADD*, *CEP55*, *PLAC8*, *TTK* and *FOS)* showed high sensitivity (0.67 to 1) in detecting treatment failure. The other markers showed low sensitivity and specificity (S3 Table) in both the groups.

HPV positivity was a good prognosticator in the overall cohort with 37% (3/8) showing treatment failure as compared to 63% (7/11) in negative patients. However, in combination with FADD, the only marker that showed significant association with HPV status, FADD^high^/HPV- patients showed a high percentage of treatment failure (60%, 6/10) as compared to FADD^low^/HPV+ patients (33%, 1/3).

**Fig 4:**
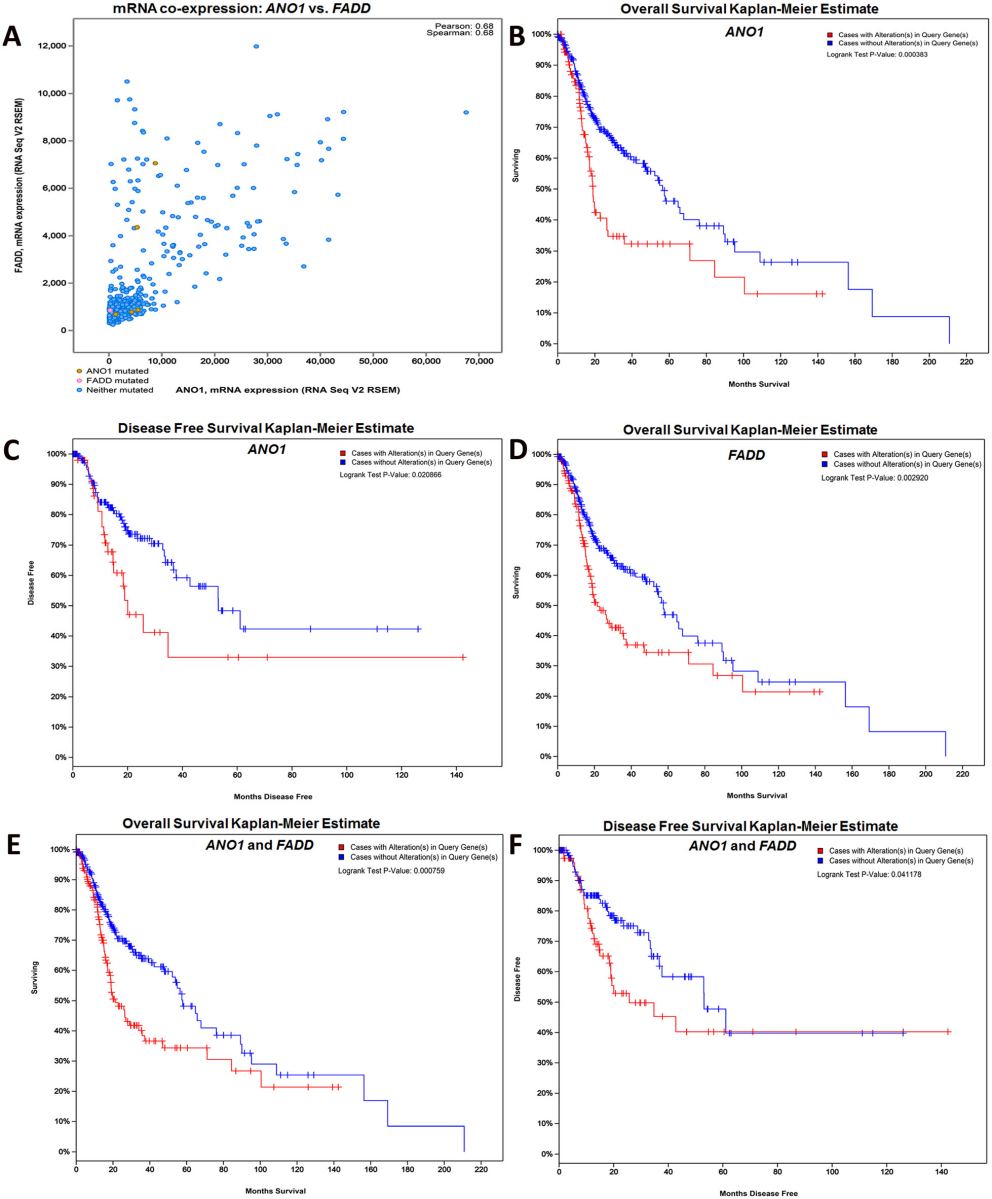
Validation with the TCGA database. The selected markers were analyzed in the TCGA database for the co-expression, overall survival and disease free survival for their significance in the HNSCC TCGA provisional study. *ANO1* and *FADD* showed highest correlation in the co-expression analysis (A) with Pearson’s and Spearman’s correlation (0.68). *ANO1* and *FADD* were further analyzed for their overall survival (OS) (B and D) and Disease free survival (DFS) (*ANO1*; C). Patients with *ANO1* over-expression showed low median survival (18.96 vs 56.44 months; p = 0.0003) and low DFS (20.04 vs 53.09 months; *p* = 0.02) when compared with the cohort without alterations (B and C). *FADD* showed association with OS wherein low median survival (21.48 vs 57.42; *p* = 0.002) was observed in patients with an upregulation of the gene (D). Both *ANO1* and *FADD* when assessed in combination, were associated with low median survival (21.48 vs 57.88; *p* = 0.0007) (E) and disease free survival (25.72 vs 53.09; *p* = 0.04) (F) in altered cases when compared to cases without alterations.

Among these genes, only *ANO1* and *FADD* showed significant association with overall survival (OS) and disease free survival (DFS) (*p*<0.05) in TCGA database. In addition, co-expression data showed that both these genes are expressed in patients with HNSCC (Spearman’s correlation = 0.68) (Fig 4A). The 109 cases with upregulated expression of *ANO1* showed low median survival as compared to the cohort without any alterations (18.96 vs 56.44 months; *p* = 0.0003) (Fig 4B). Similar association was also observed in the patients with treatment outcome (N = 217). Fifty four of these patients with increased *ANO1* expression had a low DFS (20.04 vs 53.09 months; *p* = 0.02) (Fig 4C). *FADD* showed association only with overall survival (*p* = 0.002), patients with up regulation (133/400) showed a low median survival (21.48 vs 57.42 months; *p* = 0.002) (Fig 4D). Assessment of the combined expression showed that co-expression of these two genes had a significant effect on OS (21.48 vs 57.88; *p* = 0.0007) and DFS (25.72 vs 53.09; *p* = 0.04) in HNSCC patients (Fig 4E and 4F).

## Discussion

High throughput global profiling at the transcript and protein levels provide an extensive repertoire of probable candidate biomarkers for diagnosis and prognosis of various cancers. Nevertheless, issues pertaining to the analysis of data, possible cross-study discordance and the need for selecting apt candidates for extensive patient based validation has made the clinical utilization of this data extremely challenging. In addition, the data generated from any single study using these approaches are rarely utilized completely, with only subsets of the data being analyzed in context of the specific objective for which the study is designed. Multiple *in vitro* and patient related studies have been carried out in head and neck cancer that profiles the expression differences in the tumor as compared to the tumor adjacent mucosa or the completely normal tissue [11, 25, 26]. Consequently, although a number of disease-specific biomarkers have been identified, very few have been translated to clinical application. These issues associated with global profiling studies necessitates optimal utilization of publically available databases and identification of a functionally relevant, robust subset of biomarkers that can be validated for their clinical applicability. Meta—analysis, a systematic method that enables data analyses from independent studies and integrates them using common statistical pipelines, is invaluable in this context [17]. The primary objective of this study was to identify a functionally relevant and clinically applicable subset of biomarkers for HNSCC by utilizing a meta-analysis based approach.

The meta-analyses approach has been employed to identify clinically relevant biomarkers in cancers of many sites. Studies across microarray datasets in pancreatic cancer, prostate cancer and osteosarcoma have led to the identification of novel targets as well as biomarkers [12, 13, 27]. This approach has also led to gene signatures that predict treatment outcome and relapse-free survival in breast cancers that are now in clinical use [14, 15]. Publically available databases on expression profiling of HNSCC patients were analyzed in a previous study, leading to identification of a signature for survival [28]. Another study identified six categories of HNSCC patients based on their molecular profile with the hypoxia-associated and mesenchymal sub-types being more aggressive [29]. Our study identified a subset of genes based on concordance across studies carried out using different technological platforms, thereby imparting a higher confidence value with regard to their potential clinical utility. The high percentage of genes (>80%) that were experimentally validated in terms of their trends in regulation between the microarray studies and the patient expression profiles further emphasized the significance of this approach to identify clinically relevant markers. The patient cohorts used in the study was of a smaller size but a further validation using TCGA data emphasized the clinical relevance of the selected markers. Cross-platform analysis carried out across the Affymetrix and Agilent platforms also highlighted the high percentage of discordance (>50%) among the trends of the genes identified. This discordance may be reflective of genuine biological differences owing to tumor heterogeneity across different sample cohorts or due to technical artifacts; nevertheless, this aspect further emphasizes the need for a meta-analysis based approach for marker identification.

Cross-database comparison of the 181 gene signature identified in this study with the TCGA data identified *ANO1* and *FADD* as altered with high frequency in HNSCC (>30%) and further these genes showed significant association with overall and disease-free survival. Both these genes are located on chromosome 11 (11q13), a highly amplified region in cancers [30, 31]. Anoctamin 1 (*ANO1*), a transmembrane, Ca (2+) activated Cl (-) channel is known to be specifically expressed in HNSCC (>80% of the tumors) [32] correlating with the development of distant metastasis [33]. The expression of this gene, regulated by promoter hyper-methylation, is known to provide the balance between tumor growth and metastasis [34]. Further, i*n vitro* studies have shown that *ANO1* promotes anchorage-independent growth and cell proliferation mediated by *ERK1/2* and *CCND1* (Cyclin D1) induction [35] [32]. The Fas Associated Death Domain (*FADD*), a protein involved in apoptotic signaling [36], along with additional roles in cell proliferation, has been associated with nodal metastasis, overall and disease-free survival in patients with head and neck cancers [37] [38]. Concomitant amplification of the 11q13 region in HNSCC [39] and over expression of *FADD* have, in combination, suggested this gene to be the driver of this amplicon [40]. As observed in this study, both these genes, in combination, are also identified to be predictive of prognosis in breast cancers [41]. Identification of these two genes from this cross-study/cross-platform analysis, necessitates detailed investigation as to their role in the disease and their clinical applicability as biomarkers and/or therapeutic targets.

Among the other genes validated, *ECT2* was altered in the highest number of HNSCC patients in TCGA. This cytokinesis regulator, when dysregulated, can activate Rho signaling pathways and thereby may result in malignant transformation as observed in lung adenocarcinoma and glioma [42–44]. A study in oral cancer has further implicated the gene to be involved in cellular proliferation and therefore would be of relevance in early carcinogenesis [45]. In this study, *PLAC8* and *UBE2V2* showed validation across maximum percentage of patient samples (90%). Interestingly, *PLAC8* is reported to be a candidate oncogene and is known to affect regulation of autophagy and promote ERK-dependent epithelial mesenchymal transition in colon cancers [46, 47]. However, i*n vitro* studies in other cancer cell lines have shown contradictory functions of the gene, wherein it contributes towards pro-apoptotic functions [48]. These functional differences are suggested to be due to possible splice variants of the gene, an aspect that needs to be extensively investigated in order to establish its role in the early carcinogenesis of head and neck cancer. Studies that report the role of *UBE2V2* in cancer are comparatively few, the gene is reported to be associated with poor prognosis in breast cancer [49] while the suppression of the gene in colorectal carcinoma cells is known to reverse oxaliplatin resistance [50]. Further studies are essential to elucidate its role in Head and Neck cancer.

Human papilloma virus is considered a major etiological factor in Head and Neck cancers especially in oropharyngeal cancers [51], recent evidence suggest an increasing influence in cancers of the oral cavity also [52]. Cancers with a HPV-based etiology are reported to lesser frequency of genetic changes [53, 54], in this study although the overall differential expression pattern of the genes in the patients did not correlate to HPV status, *FADD* showed a significant association. Majority of the HPV+ patients showed a downregulation of *FADD*, in concordance with reports that HPV oncoprotein E6 binds to *FADD* and subsequently leading to a degradation of the protein [55]. This is considered a protective mechanism against apoptosis. In combination with the association of *FADD*/HPV positive patients with recurrence and prognosis, these evidences indicate contrasting roles for the gene that is based on the etiology of the cancer, further *in vitro* studies and patient validation can establish the same.

A surprising aspect of the study is the absence of the reported and known players in head and neck cancer, Epidermal Growth Factor Receptor (*EGFR)*, Tumor Protein p53 (*TP53)* and *Ras* from the statistically significant list of differentially expressed genes. Previous review of transcriptomic profiling studies in head and neck cancer also showed that these major players have been absent from most transcriptomic studies [56]. This might suggest that the differences at transcript level expression of these genes are not as significant in the patients with the disease compared to the mutation based and protein level differences. On the other hand, the other known classes of genes reported previously such as the collagen family, Serpins, *FN1*, Cyclin dependent kinases are well represented in our cohort. Documentation of new molecules such as *ECT2* and *PLAC8*, not extensively reported in HNSCC previously, further indicates that the meta-analysis based approach can enable identification of novel candidate biomarkers and therapeutic targets.

Analysis of the interaction networks between the statistically significant concordant genes showed two major clusters with many genes as cross connectors. *FOS* and *MYC*, the well-known molecules involved in cell proliferation and differentiation were prime among them. The FOS family of genes is known to be correlated with increased aggressiveness of the tumors, their metastatic nature and thereby conferring poor prognosis in HNSCC [25, 57, 58]; a similar correlation was obtained from this study. *MYC* along with its co-amplified partner, *CDK1* are reported to be correlated with survival and prognosis in head and neck cancers [59–61]. The significance of this network and the cross connecting nodes needs to be further investigated to understand their biological significance.

This study has hence identified a list of biomarkers of head and neck cancer with high confidence in terms of their functional and biological relevance and potential clinical applicability. Identification of these markers, concordant across multiple studies and different technologies, suggest their strong relevance to HNSCC. This study did not look into the site-specific variations that might be relevant in the biology of the disease; validation in larger and well annotated patient cohorts might provide insights into their specific roles in the process. In addition, similar meta-analysis based approaches towards identification of markers of resistance/response to chemotherapy in the patients will be extremely beneficial. Studies in this direction are currently ongoing in our group.

## Supporting Information

S1 Fig **(A) Gene Ontology analysis of the concordant gene list (PANTHER database)**. In molecular function category (a), the binding category (28.5%) showed maximum number of gene entities. Similarly in biological process category (b), cellular (20.10%) and metabolic process (18.8%) while in cellular components category (c), the cell parts and extracellular region showed maximum gene entities (24.40%). **(B) Cross comparison with TCGA**. The concordant, significant gene entities were analyzed for mutation, copy number variation (CNV) and gene expression alteration status in TCGA HNSCC patient cohort. Twenty out of the total entities that showed alteration in ≥15% of the patients are shown in the figure. The genes *ECT2*, *ANO1* and *TP63* showed alteration in highest proportion (>35%) of the patients. These gene entities showed maximum alteration in the CNV and at the gene expression levels.
(TIF)

S2 Fig Efficiency of the Primers. The efficiency of the selected genes (N = 9) is represented (A-I). The standard curves (Cp vs. Log concentration) were generated with a set of serially diluted cDNA concentrations and the slope generated to calculate efficiency. (A-I). The Efficiency ranged from 1.95 to 2.12 for all the primers.
(TIF)

S3 Fig Selection of Reference Genes. The efficiency of the selected reference primer are represented; the values ranged from 1.86–2.01 (**A-D**). Analysis of the expression profile of these genes in the RefFinder, identified *18SRNA* and *RPLP0* as the most stable reference genes while *GAPDH* and *GUS* were categorized as less stable (**E**). *18SRNA* and *RPLP0* were used for relative quantification of the target genes.
(TIF)

S4 Fig Correlation of Markers with HPV. The expression of p16 in the Head and neck cancer samples was represented as no staining, score = 0 (A), weak staining, score = 1 (B), moderate, score = 2 (C) and strong staining, score = 3 (D). Correlation of this status with the different markers indicated a statistically significant (p = 0.03) association with the expression pattern of *FADD* (E). The arrow mark indicates the p16 positive regions.
(TIF)

S1 File (A) List of concordant, differentially expressed genes obtained from Affymetrix platform (B) List of concordant pathways obtained from Affymetrix platform (C) List of concordant, differentially expressed genes from Agilent platform (D) List of concordant pathways obtained from Agilent platform.
(XLSX)

S2 File (A) List of concordant, differentially expressed genes obtained from cross platform analysis (B) List of concordant pathways obtained from cross platform analysis.
(XLSX)

S3 File Correlation of the concordant genes with the TCGA Database (mutation, copy number variation and expression). (XLSX)

S1 PRISMA Checklist (DOC)

S1 Table List of primers. (XLSX)

S2 Table Clinical characteristics of the Patients. (XLSX)

S3 Table Association of the selected markers with recurrence/re-recurrence in the two patient cohorts. (XLSX)
