# Supplementary figures and images for: Meta-Analyses of Microarray Datasets Identifies ANO1 and FADD as Prognostic Markers of Head and Neck Cancer
Source: PLoS One. 2016 Jan 25;11(1):e0147409. doi: 10.1371/journal.pone.0147409 (PMC4726811; doi:10.1371/journal.pone.0147409)

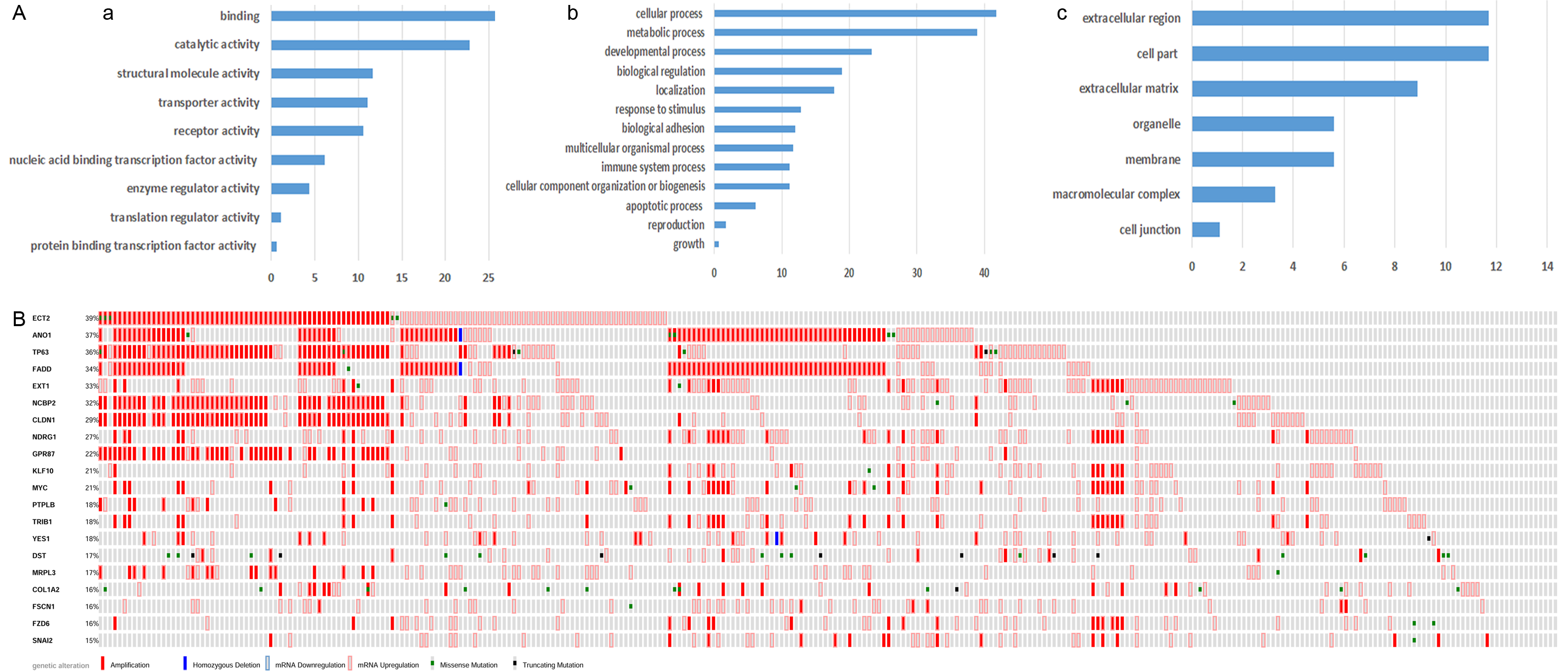

Supplement: S1 Fig — (A) Gene Ontology analysis of the concordant gene list (PANTHER database). In molecular function category (a), the binding category (28.5%) showed maximum number of gene entities. Similarly in biological process category (b), cellular (20.10%) and metabolic process (18.8%) while in cellular components category (c), the cell parts and extracellular region showed maximum gene entities (24.40%). (B) Cross comparison with TCGA. The concordant, significant gene entities were analyzed for mutation, copy number variation (CNV) and gene expression alteration status in TCGA HNSCC patient cohort. Twenty out of the total entities that showed alteration in ≥15% of the patients are shown in the figure. The genes ECT2, ANO1 and TP63 showed alteration in highest proportion (>35%) of the patients. These gene entities showed maximum alteration in the CNV and at the gene expression levels. (TIF) [file pone.0147409.s001.tif]

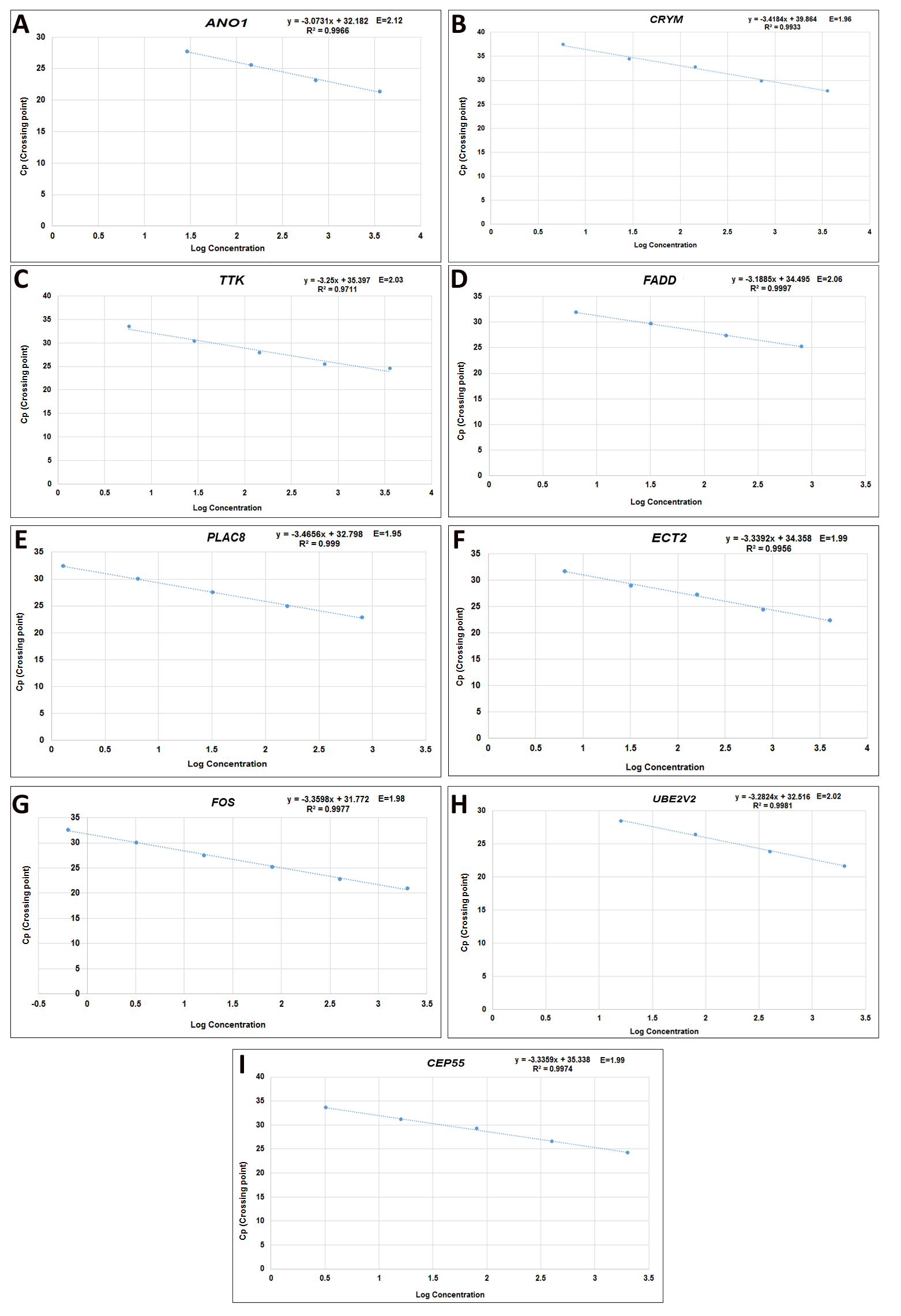

Supplement: S2 Fig — The efficiency of the selected genes (N = 9) is represented (A-I). The standard curves (Cp vs. Log concentration) were generated with a set of serially diluted cDNA concentrations and the slope generated to calculate efficiency. (A-I). The Efficiency ranged from 1.95 to 2.12 for all the primers. (TIF) [file pone.0147409.s002.tif]

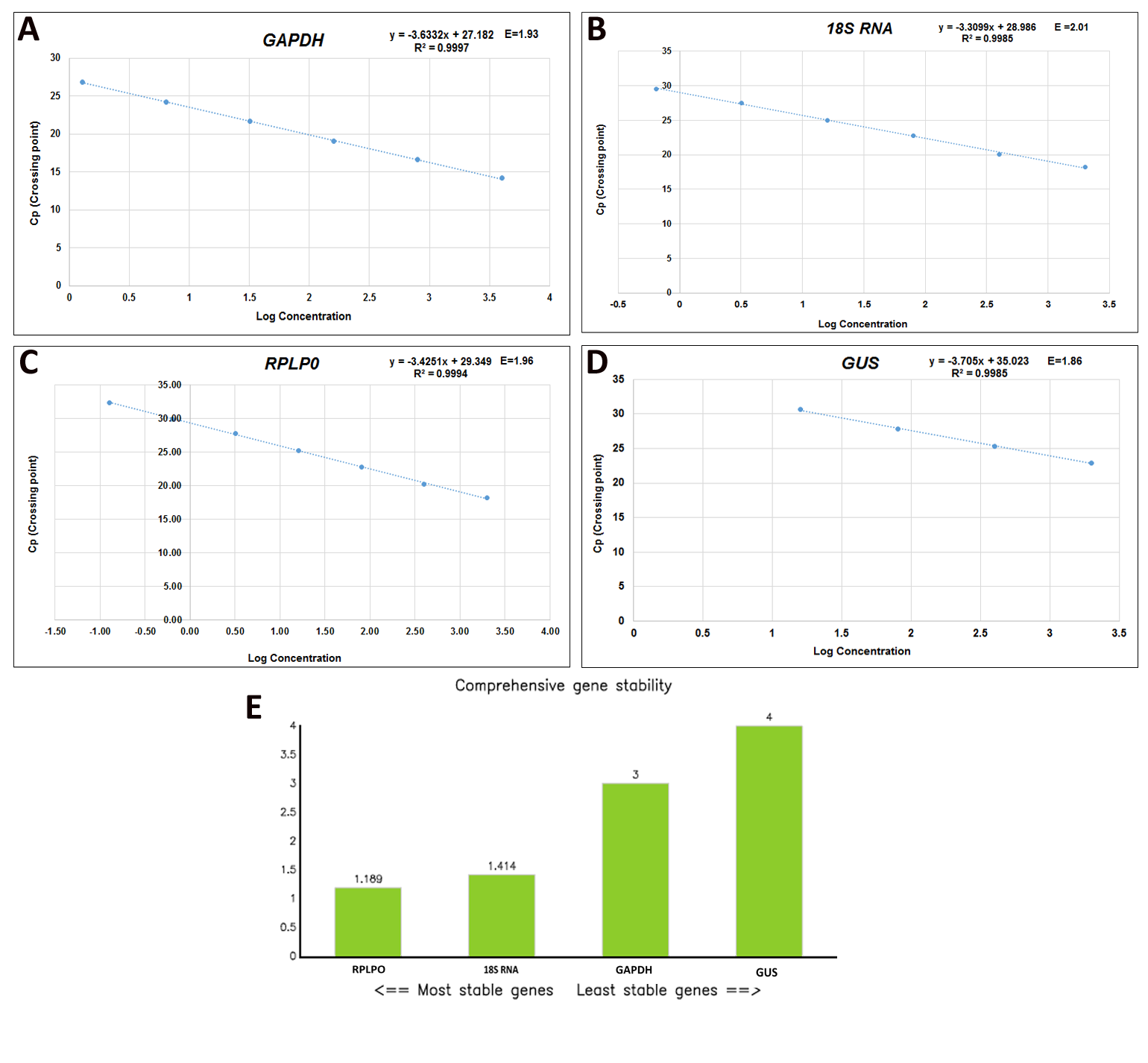

Supplement: S3 Fig — The efficiency of the selected reference primer are represented; the values ranged from 1.86–2.01 (A-D). Analysis of the expression profile of these genes in the RefFinder, identified 18SRNA and RPLP0 as the most stable reference genes while GAPDH and GUS were categorized as less stable (E). 18SRNA and RPLP0 were used for relative quantification of the target genes. (TIF) [file pone.0147409.s003.tif]

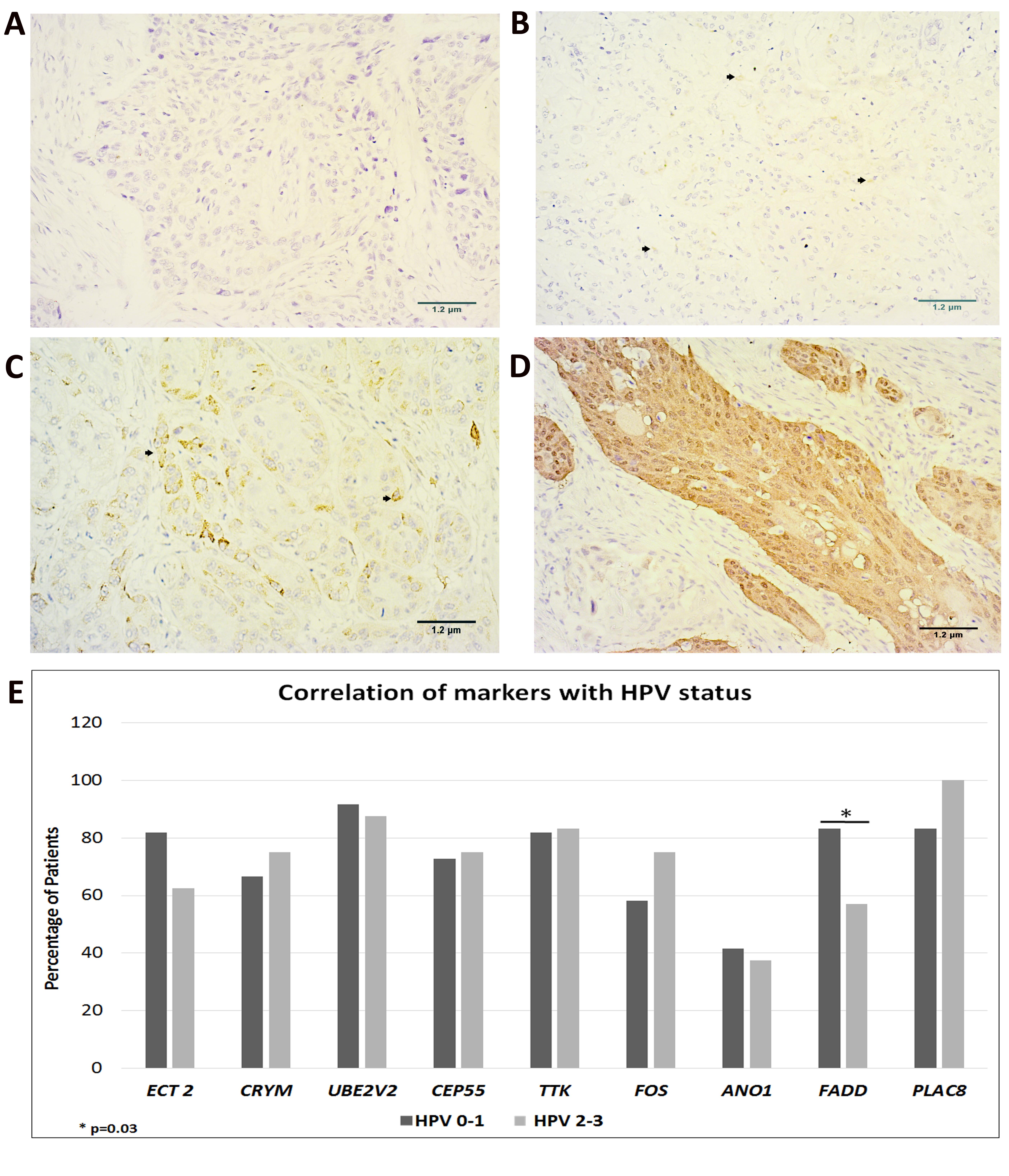

Supplement: S4 Fig — The expression of p16 in the Head and neck cancer samples was represented as no staining, score = 0 (A), weak staining, score = 1 (B), moderate, score = 2 (C) and strong staining, score = 3 (D). Correlation of this status with the different markers indicated a statistically significant (p = 0.03) association with the expression pattern of FADD (E). The arrow mark indicates the p16 positive regions. (TIF) [file pone.0147409.s004.tif]
